# Comparative transcriptomics reveals different profiles between diflubenzuron‐resistant and ‐susceptible phenotypes of the mosquito *Culex pipiens*

**DOI:** 10.1002/ps.8710

**Published:** 2025-02-12

**Authors:** Mastrantonio Valentina, Liberati Franco, Castrignanò Tiziana, Lucchesi Valentina, Urbanelli Sandra, Bellini Romeo, Vontas John, Porretta Daniele

**Affiliations:** ^1^ Department of Environmental Biology Sapienza University of Rome Rome Italy; ^2^ Department of Ecological and Biological Sciences Tuscia University Viterbo Italy; ^3^ Department of Medical and Veterinary Entomology Centro Agricoltura Ambiente ‘G. Nicoli’ Bologna Italy; ^4^ Institute of Molecular Biology and Biotechnology, Foundation for Research and Technology‐Hellas Heraklion Greece; ^5^ Pesticide Science Laboratory, Department of Crop Science Agricultural University of Athens Athens Greece

**Keywords:** insecticide resistance, insect growth regulators, mosquitoes, RNA‐Seq, vector species

## Abstract

**Background:**

Chitin‐synthesis inhibitors (CSIs) represent a major tool in vector control. The intensive use of these compounds has led to the evolution of resistance against several CSIs, including diflubenzuron (DFB). DFB resistance has been associated to a target‐site mechanism; however, studies investigating the gene expression profile of resistant phenotypes are limited, preventing a full understanding of DFB resistance. Here, we analyzed the constitutive gene expression of susceptible and DFB‐resistant individuals of the mosquito *Culex pipiens,* a major disease vector in temperate areas.

**Results:**

Comparative gene expression analysis between susceptible and DFB‐resistant individuals identified 527 differentially expressed genes (i.e., 432 up‐regulated and 95 down‐regulated genes). Among the up‐regulated genes, 87 genes belong to gene families associated with insecticide resistance in arthropods, such as cytochrome P450s, glutathione‐S‐transferases, UDP‐glucuronosyltransferases, heat shock proteins and cuticular proteins (CPs). Interestingly, the CP transcripts were the most abundant among up‐regulated genes (73 of 87), and furthermore they constitute 11 of the 20 most over expressed genes. The enrichment of transcripts associated with cuticle synthesis was also identified by the Gene Ontology (GO) enrichment analysis.

**Conclusions:**

Adaptation to insecticides can involve transcriptional changes in genes encoding for multiple defense mechanisms. Our results identified the over‐expression of transcripts associated with detoxification and cuticle synthesis in DFB‐resistant individuals of *Cx. pipiens*. Multiple mechanisms, beyond the known target‐site mechanism, may therefore contribute to the DFB‐resistant phenotype. Together these findings corroborate the complexity underpinning the resistant phenotypes and provide important information for the implementation of effective control strategies against mosquito vectors. © 2025 The Author(s). *Pest Management Science* published by John Wiley & Sons Ltd on behalf of Society of Chemical Industry.

## Graphical abstract

Comparative gene expression analysis between susceptible and diflubenzuron‐resistant individuals of the mosquito *Culex pipiens* revealed transcriptional changes of multiple genes associated with insecticide detoxification and cuticle synthesis.

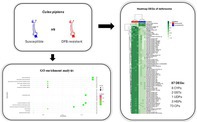

## 1 INTRODUCTION

Mosquito‐borne diseases (MBDs) represent one of the most important public health concerns worldwide. About 390 million people are infected each year with arbovirus carried by mosquitoes such as dengue, Zika, West Nile, and chikungunya.^1^, ^2^ To date, the control of mosquito populations remains the main strategy for limiting the transmission of MBDs.

The use of insecticides still represents a common strategy to attempt the suppression of vector populations.^3^ Neurotoxic compounds, such as organophosphates and pyrethroids, have been major classes used to control mosquitoes over the last decades.^4^ However, their constant and intensive use has led to the emergence of negative environmental effects, such as toxicity on non‐target species and the evolution of insecticide resistance.^5^, ^6^, ^7^, ^8^, ^9^ Vector control programs are therefore continuously looking for valuable alternatives to counteract these drawbacks.

Chitin‐synthesis inhibitors (CSIs) have been identified as an important tool in vector control due to their low toxicity to off‐target fauna.^10^, ^11^ Among these compounds, diflubenzuron (DFB) has been largely employed against mosquitoes, especially in Europe.^11^ This larvicide acts against chitin synthesizing organisms by directly interfering with the chitin synthase 1 (CHS1) enzyme, causing abortive molting and defects in egg hatching.^12^, ^13^, ^14^ Recently, concern about the effectiveness of DFB in mosquito control have emerged, because target‐site resistance was detected in the common‐house mosquito *Culex pipiens*.^15^, ^16^

*Culex pipiens* is a widespread species across the Mediterranean region, where beside nuisance, it is also responsible of the recent West Nile virus outbreaks.^17^, ^18^ In 2015, the first evidence of DFB resistance was found in some Italian *Cx. pipiens* populations from the Emilia‐Romagna region.^15^ Molecular analyses revealed that the resistant phenotype was associated to three non‐synonymous mutations in the position 1043 of the CHS1 gene, that changed the amino acid isoleucine (i.e., I1043) with leucine, methionine or phenylalanine (i.e., I1043L, I1043M, I1043F, respectively).^15^, ^16^ Subsequently, functional analyses showed that these mutations were associated with the extremely high levels of resistance, with the I1043M and I1043F mutations reaching a resistance ratio (RR) up to 15 000‐fold.^14^, ^15^, ^16^

Since the first detection in Italy, studies about DFB resistance have enhanced the understanding regarding the distribution of resistant mutations across the Mediterranean basin, as well as about the phenotypic characteristics associated with the resistant genotypes. Resistant mutations have been documented in multiple areas beyond Italy, including France, Greece, and Turkey.^16^, ^19^, ^20^, ^21^, ^22^ Interestingly, in depth analysis of the homozygous I1043M‐resistant individuals showed that they harbor a thicker cuticle and a higher chitin content than susceptible individuals.^23^

In several species, phenotypic differences between resistant and susceptible individuals have been associated with constitutive changes in gene expression. For example, constitutive over‐expression of detoxifying gene families has been observed in individuals resistant to neurotoxic insecticides. This suggests a role for metabolic detoxification in resistance, and in some cases functional genetic approaches allowed to reveal which genes are involved in resistance; this is the case of cytochrome P450 monooxygenases and adenosine triphosphate (ATP)‐binding cassette (ABC) transporters.^5^, ^24^, ^25^, ^26^, ^27^, ^28^, ^29^ Beyond detoxifying genes, over‐expression of genes encoding for cuticle synthesis has been documented and it resulted in cuticular changes between resistant and susceptible individuals (i.e., penetration resistance).^30^ This evidence highlights the complexity often underpinning resistant phenotypes and suggests the need to investigate gene expression to fully understand insecticide resistance. Contrary to other insecticides for which the expression profile in resistant individuals has been widely elucidated, the whole gene expression pattern underlying resistant phenotype to CSIs in mosquitoes remains understudied. Here, we aim to contribute to filling this gap by analyzing the constitutive gene expression of susceptible and DFB‐resistant individuals of the mosquito *Cx. pipiens*.

## 2 MATERIALS AND METHODS

### 2.1 Mosquito samples

*Culex pipiens* individuals used to generate the dataset were obtained from colonies established from field populations as described in Lucchesi *et al*.^23^ The susceptible colony (*Cp‐*S) was homozygous for the wild‐type allele I1043 (*Cp*‐S‐I1043), while the resistant colony was homozygous for the resistant allele I1043M (*Cp*‐R‐I1043M). Bioassays showed significant different levels of DFB resistance with a LD_50_ [i.e., the lethal dose killing half (50%) of the mosquitoes tested] of 0.005 mg L^−1^ (0.002–0.008) and 45.03 mg L^−1^ (13.06–60.66), respectively, corresponding to a RR = 9000.^23^

Larval stages of both colonies were reared in plastic trays filled with 2 L of spring water and daily feed with 0.85 mg per larva of fish food (Tetra® Goldfish Granules). After emergence, adults were put in 45 cm × 45 cm × 45 cm insect‐rearing cages (Bugdorm®; Watkins & Doncaster, Leominster, UK) and daily fed with 10% sucrose solution *ad libitum*. Blood meal was provided to females for egg maturation by using the Hemotek membrane feeding‐system (Hemotek Ltd, Blackburn, UK). Adult and immature stages were reared in a thermostatic chamber under controlled conditions of temperature (*T* = 26 ± 1 °C), relative humidity (RH = 70%) and photoperiod (L:D = 16 h:8 h light/dark).

Mosquitoes belonging to the fifth generation were used from both colonies for constitutive RNA‐sequencing (RNA‐Seq). To complete the RNA‐Seq four trays filled with 2 L of spring water and 200 first‐instar larvae (L_1_ larvae) were set up for each strain. The larvae were maintained as described earlier until the fourth instar; then four pools of ten larvae were randomly collected from each tray and immediately stored in RNA Later® at −80 °C until RNA extraction.

### 2.2 RNA extraction, library preparation, and sequencing

RNA extraction was performed from whole‐body pooled larvae of *Cx. pipiens* by using the NucleoSpin RNA Plus XS kit (Macherey‐Nagel, Düren, Germany), according to the manufacturer's instructions. A step including a DNase treatment was also performed during RNA extraction. Four pools were used for the *Cp‐*S and *Cp*‐R‐I1043M colony, respectively (Supporting Information Table S1). The integrity and quality of the total RNA were assessed by Qubit fluorimetry and the 5200 Fragment Analyzer (Agilent Technologies, Santa Clara, CA, USA). Library preparation and sequencing were performed by the Polo GGB (Polo d'Innovazione di Genomica, Genetica e Biologia), Perugia, Italy (http://www.pologgb.com/). Libraries were prepared from high quality total RNA (1.5–2.0 μg) using the QIAseq Stranded mRNA Library Kit and following the manufacturer's instructions. The sequencing was done as a 2 × 75 bp paired end run using the Illumina® NextSeq 550 system. On average 70.8 million reads for each library were generated and deposited at the National Center for Biotechnology Information (NCBI) Sequence Read Archive (project ID PRJEB47420).

### 2.3 Transcriptomics analyses

The workflow of transcriptomic analyses is described in the Supporting Information Fig. S1. The links to the files produced are provided in Table S2 and are available on the Figshare archive. The raw sequences obtained were processed using Salmon software version 1.5.1,^31^ a transcriptome‐wide quantification tool that enhances the precision of abundance estimations for downstream differential expression analyses. We ran Salmon in quasi‐mapping mode, using both the raw sequencing reads and the index of the *de novo* transcriptome of *Cx. pipiens*,^32^ which is publicly accessible via Figshare (Table S2, datafile 2). After obtaining transcript quantification with Salmon, we proceeded with the analysis of differentially expressed genes (DEGs) using the Iguaner pipeline,^33^ which is based on the DESeq2 tool.^34^ Iguaner generates DEG lists for all experimental conditions of interest, produces various visualization plots [principal component analysis (PCA) plots, volcano plots, heatmaps], and performs gene set enrichment analysis.

For the annotation of the identified DEGs, we employed two complementary methods: homology‐based and functional annotation. Diamond software version 2.0.11^35^ was used for homology annotation using standard parameters. We performed sequence alignments with Diamond (blastp function), by mapping the predict open reading frames (ORFs) against the Nr, SwissProt, and TrEMBL databases, producing high‐accuracy homology‐based annotations for each DEG sequence using HPC‐T Annotator software.^36^ EggNOG Mapper (version 2)^37^ was utilized for functional annotation. We compared DEG sequences with EggNOG Mapper against the comprehensive homologous groups cataloged in the EggNOG database, enabling functional annotation based on three key databases: Gene Ontology (GO), Kyoto Encyclopedia of Genes and Genomes (KEGG), and Clusters of Orthologous Groups (COG). A GO Slim using the software QuickGO^38^ was also performed by using the predefined GO Slim of *Drosophila melanogaster*. Enrichment statistics and hypergeometric testing were additionally performed on the subset of DEGs belonging to detoxifying enzymes (i.e., 98 up‐regulated genes and 8 down‐regulated genes).

## 3 RESULTS

### 3.1 RNA‐sequencing

Sequencing of susceptible and resistant strains of *Cx. pipiens* yielded 587 164 752 of paired‐end raw reads, ranging from 49.6 to 83.7 million reads per library. The GC‐content percent of samples ranged from 40% to 48%, and the mapped ratio of reads against the *de novo* reference assembly was higher than 80% for all samples (Table S1).

### 3.2 Differential expression analysis

**Figure 1:**
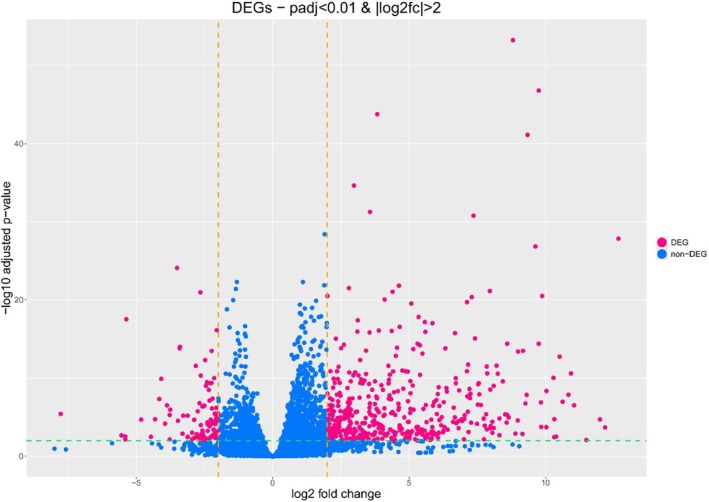
Volcano plots of gene expression profiles of diflubenzuron susceptible and resistant *Culex pipiens* mosquitoes (*P*_adj_ ≤ 0.01 and log_2_FC > 2).

**Figure 2:**
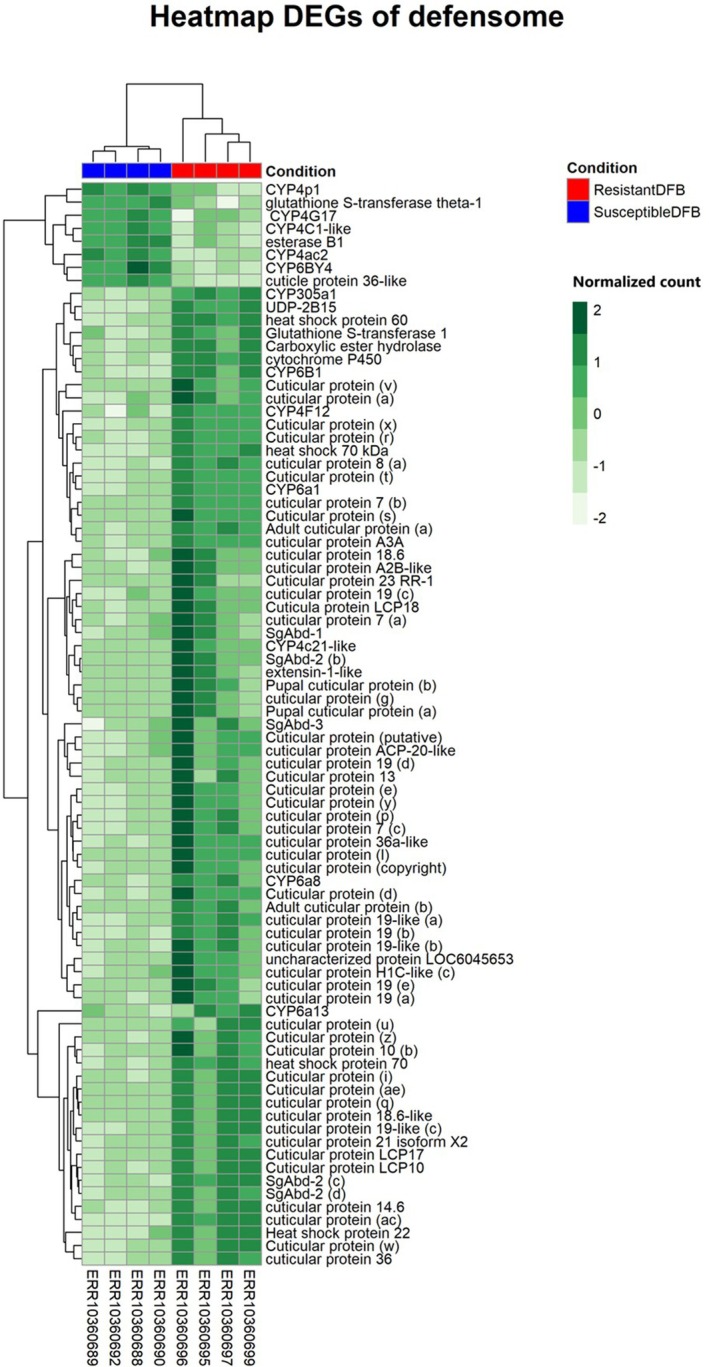
Heatmap of defensome genes. Heatmap showing differential gene expression between diflubenzuron (DFB)‐susceptible and DFB‐resistant *Culex pipiens* mosquitoes, sorted by log_2_FC value and with colors indicating normalized counts. Only the expression of genes potentially associated with insecticide defense/resistance is shown.

Comparative analysis of gene expression between the susceptible and DFB‐resistant strains of *Cx. pipiens* identified a total of 527 DEGs [adjusted *P*‐value (*P*_adj_) ≤ 0.01 and log_2_fold change (log_2_FC) > 2], with 432 up‐regulated genes and 95 down‐regulated genes (Fig. 1 and Table S3). PCA of transcriptome‐wide normalized gene expression counts showed a clear separation between susceptible and resistant groups along principal component one (PC1), explaining 60% of the variance, and PC2, explaining 16% of the variance (Fig. S2). The PCA values, derived by DESeq2, are already normalized to minimize the influence of technical differences and balance variance. Among the up‐regulated genes, 87 genes belong to gene families associated to insecticide resistance in arthropod species (Fig. 2, Tables 1 and S4). In particular, we found a significant increase in the expression of eight cytochrome P450s (CYP4c21, CYP6a8, CYP305a1, CYP4V2, CYP6a13, CYP4d2, CYP6b1, and CYP4f12), two glutathione‐S‐transferases (GSTs) (GST1, GSTD2), one UDP‐glucuronosyltransferase (UDP2B15), three heat shock proteins (HSP70, HSP60, HSP22), and cuticular proteins (CPs). Interestingly, genes belonging to CPs were the most abundant among up‐regulated genes, with 73 over‐expressed genes. Similarly, members of detoxifying families were also detected among down‐regulated genes, with the cytochrome P450 family as the most represented (Fig. 2, Tables 1 and S4). Notably, CP members were among the top 20 over‐expressed genes in our comparisons (11 of 20). Other genes related to Osiris protein families, fibrinogen C‐terminal domain‐containing protein and proline‐rich protein, were among the top 20 genes over‐expressed in the R‐S comparisons (log_2_FC ~ 9.34–12.67) (Table S3).

**Table 1:** Number of up‐ and down‐regulated genes belonging to the known gene families involved in insecticide resistance

Defensome families	Up‐regulated genes	Down‐regulated genes
Cytochrome P450	8	5
Esterases	—	1
Glutathione S‐transferases	2	1
UDP‐glucuronosyltransferases	1	—
Heat shock proteins	3	—
Cuticular proteins	73	1
Total	87	8

*Note*: a log_2_FC > 2 was used to identify the differentially expressed genes.

### 3.3 Functional annotation

Differentially expressed transcripts with GO annotation were assigned to three functional classes: biological processes, cellular components and molecular functions (Fig. S3). The most represented up‐regulated transcripts in biological processes were cellular process (GO: 0009987); in cellular components they were extracellular region (GO: 0005576); in molecular functions they were binding (GO: 0005488). The most represented down‐regulated transcripts in biological processes were metabolic process (GO: 0008152); in cellular components they were cell part (GO: 0033643); in molecular functions they were binding (GO: 0005488) (Fig. S3). By using GO Slim annotation, the most represented categories of up‐regulated gene product in biological processes were lipid metabolic process (GO: 0006629) and monoatomic ion transport (GO: 0006811); in cellular components they were extracellular matrix (GO: 0031012); in molecular functions they were structural constituent of cuticle (GO: 0042302). The most represented categories of down‐regulated gene were monoatomic ion transport (GO: 0006811) in biological processes; endoplasmic reticulum in cellular component (GO: 0005783); oxidoreductase activity (GO: 0016491) (Table S5). GO enrichment analysis for up‐regulated transcripts identified six GO terms in biological processes and molecular functions, and one term in the cellular component class (Tables S6 and S7). No GO terms were identified for down‐regulated transcripts.

## 4 DISCUSSION

In recent decades several studies have revealed that the whole genome expression profile of resistant and susceptible individuals can significantly differ. Here, we have analyzed the constitutive gene expression of susceptible and DFB‐resistant individuals of the mosquito *Cx. pipiens*. Our differential expression analysis identified several transcripts associated with detoxification enzymes and cuticle synthesis over‐expressed in DFB‐resistant larvae.

Metabolic detoxification is one of the main mechanisms of insect adaptations to insecticide treatments.^5^, ^6^, ^7^, ^8^, ^9^, ^39^ Enzymes belonging to cytochrome P450s, GSTs, and carboxylesterases (CCEs) are widely associated with insecticide resistance because they allow the chemical modification and/or sequestration of the toxic molecules.^39^ In *Cx. pipiens*, we found a significant increase in the expression of cytochrome P450 genes from the CYP3, CYP4 and CYP6 families, which are known to be involved in insecticide resistance in many species, including mosquitoes.^40^ Other metabolic genes, including two GSTs and one UDP‐glucuronosyltransferase, were also over‐expressed in resistant *Cx. pipiens* individuals. The up‐regulation of cytochrome P450 and GSTs was demonstrated in multiple species against CSIs. Comparative transcriptomic analysis of lufenuron‐resistant and ‐susceptible strains of *Spodoptera frugiperda* showed the up‐regulation of genes encoding for cytochrome P450 enzymes in resistant individuals.^41^ The role of CYPs in lufenuron detoxification has also been suggested in *D. melanogaster*, as CYP12A4 was up‐regulated in resistant individuals.^42^ Sonoda and Tsumuki^43^ also found that one GST gene, GST3, was involved in the detoxification of chlorfluazuron, which led to the development of chlorfluazuron resistance in the diamondback moth *Plutella xylostella*. Finally, the up‐regulation of cytochrome P450s and GSTs was also reported in *Tribolium castaneum* exposed to DFB, which corroborates their association with the xenobiotic detoxification.^44^ Likewise, a major role of UDP‐glucuronosyltransferases was also documented in insecticide resistance. For example, UDP‐glucuronosyltransferase‐mediated resistance was detected in the flower thrips *Frankliniella occidentalis* resistant to spinosad.^45^ In *Spodoptera litura*, the silencing of UDP‐glucuronosyltransferase genes enhanced the sensitivity of larvae against indoxacarb, suggesting an important role in indoxacarb resistance.^46^ In mosquitoes, as well, the inhibition of UDP‐glucuronosyltransferases restored susceptibility to pyrethroids and DDT in the *Anopheles* malaria vectors, providing clear evidence of the association of UDP‐glucuronosyltransferases with pyrethroid resistance.^47^

Beyond detoxifying gene families, genes involved in the cuticle synthesis were also up‐regulated in resistant individuals of *Cx. pipiens*. In insects, the cuticle is made up by proteins and chitin, that are covalently bound to form a chitin‐protein complex.^48^, ^49^ In recent years, changes in cuticular thickening and composition due to the over‐expression of cuticle‐related genes have been found in pyrethroid‐ and organochlorine‐resistant strains.^30^ In *Anopheles gambiae* from West Africa, for example, a thickening of the cuticle layers due to the over‐expression of a suite of CPs was found in individuals resistant to permethrin, deltamethrin, and DDT.^50^ Relatively to DFB, the up‐regulation of genes involved in the cuticle synthesis has been recently documented in the fall webworm *Hyphantria cunea*.^51^ In *Cx. pipiens*, we found 73 CPs over‐expressed in resistant individuals, and among them some were also listed among the 20 most over‐expressed genes. Accordingly, GO enrichment analysis identified six GO terms in biological processes and molecular functions, and one term in the cellular component class associated with the cuticular pathways (Table S6). Notably, we also found the over‐expression of the *laccase 2* gene in DFB‐resistant individuals. This enzyme contributes to cuticle sclerotization by mediating the cuticle protein cross‐linking.^52^, ^53^, ^54^ Recently, the up‐regulation of this gene has been reported in resistant species showing cuticle modifications.^30^ For example, in *Cx. pipiens pallens*, the over‐expression of the *laccase 2* gene was associated with the extra sclerotized cuticle found in individuals resistant to the pyrethroid fenvalerate.^55^ Therefore, the whole expression pattern observed in *Cx. pipiens* suggest the possible occurrence of cuticle‐based mechanisms in DFB resistance. Notably, a thicker cuticle and a higher chitin content were found in DFB‐resistant *Cx. pipiens* individuals compared to susceptible individuals as expected if a penetration resistance occurs.^23^

The occurrence of multiple resistance mechanisms has been reported in several insect species and insecticide classes over the years.^39^ However, how these different mechanisms interact (i.e., additively, synergistically, or even antagonistically) and what their relative contribution is to the resistant phenotype remain largely overlooked.^56^ Although functional analyses will be required to shed light about the relative role of specific genes,^57^, ^58^ our findings support the idea that in *Cx. pipiens*, multiple mechanisms simultaneously contribute to the resistant phenotype within the same population. Therefore, this system could be useful to investigate the relative contribution of target‐site, metabolic and cuticular mechanisms to the resistant phenotype. Interestingly, among the 20 most expressed genes we found fibrinogen C‐terminal domain‐containing protein, proline‐rich protein and members belonging to Osiris protein families, which are involved in insect immunity/toxicity response.^59^, ^60^, ^61^ These results support the view of an integrated defense system (IDS) where the same organs, pathways and signaling molecules are involved against toxins, predators and pathogens.^62^ Future studies explicitly investigating this issue will help us to better understand how insects cope with abiotic and biotic pressures. Finally, important implications relative to control strategies also emerge based on our results. The over‐expression of different detoxifying genes and the occurrence of cuticular modifications have been reported against many insecticides in arthropod species, including pyrethroids, organochlorines and insect growth‐regulators.^39^ This suggests that in *Cx. pipiens* a broader resistance to other chemical classes could occur as a consequence of its expression profile, hampering the efficacy of the common control practices, including the management of resistance throughout active ingredients' rotations. Notably, sensory appendage proteins were also found over‐expressed in *Cx. pipiens*. These chemosensory proteins (CSPs) have been shown to be induced by insecticide exposure in arthropods^63^, ^64^, ^65^ and to confer pyrethroid resistance in mosquitoes.^66^ Along with this, a fitness advantage of the I1043M mutation could be associated with DFB resistance in *Cx. pipiens*, which would favor the spread of DFB resistance. Although insecticide resistance has been frequently associated to fitness costs^67^ in a recent study, Douris *et al*.^14^ compared the fitness parameters of different *Drosophila* lines and showed that the fecundity of the I1042M‐resistant flies (equivalent to the I1043M in *Cx. pipiens*) was higher than in wild‐type individuals. Whether these aspects occur in *Cx. pipiens* need to be investigated in future studies to design effective management strategies limiting insecticide resistance.

## CONFLICT OF INTEREST STATEMENT

The authors declared no conflict of interest.

## AUTHOR CONTRIBUTIONS

VM, DP, SU, and JV conceived and designed the study. VL and RB performed the sampling; VL performed the sample preparation and RNA extraction; TC and FL performed the bioinformatic analysis; VM and DP wrote the initial draft of the manuscript. All authors contributed critically to interpretation of results, edited manuscript drafts, and approved the final version.

## Supporting information


**Figure S1.** Bioinformatic workflow for transcriptomic analysis in *Culex pipiens* to identify differentially expressed genes (DEGs) associated with diflubenzuron (DFB) resistance. The process began with the analysis of both raw sequencing data (FASTQ) and a *de novo* transcriptome (unigenes) used as a reference (Mastrantonio *et al*., 2024). Transcripts were quantified using Salmon, both for transcriptome indexing and transcript quantification. Using the phenotype file (Phenodata.tsv) as input, the DEG list was generated by applying Iguaner (a tool based on DESeq2) and comparing DFB‐resistant and DFB‐susceptible samples. The results of DEGs were visualized as heatmaps and volcano plots. Open reading frames (ORFs) of DEGs were predicted using TransDecoder. Functional annotation was performed using EggNOG Mapper, and homology annotation was done with Diamond against databases (NR, Swiss‐Prot, and trEMBL). Outputs included functionally and homologously annotated DEGs, as well as functional enrichment results (e.g., gene set enrichment), also represented graphically.

**Figure S2.** Principal component analysis (PCA) shows the samples in the 2D plane spanned by their first two principal components. This type of plot is useful for visualizing the overall effect of experimental covariates and batch effects.

**Figure S3.** Presentation of Gene Ontology (GO) classification. Histograms show the number of the differentially expressed transcripts assigned to GO terms – up‐regulated (A) and down‐regulated (B) – within three functional classes: biological processes, cellular components, and molecular functions.

**Table S1.** Summary data of the eight libraries deposited in the European Nucleotide Archive (ENA, BioProject: PRJEB47420). The sample code, strain condition, and the number of raw and trimmed reads per sample are shown.

**Table S2.** Overview of produced data files and their access on Figshare.

**Table S3.** List of annotated DEGs between susceptible and resistant *Culex pipiens* mosquitoes. For each transcript, the expression value, significance, annotation and identifier are reported.

**Table S4.** List of annotated DEGs between susceptible and resistant *Culex pipiens* mosquitoes belonging to the defensome families. For each transcript, the expression value, significance, annotation, and identifier are reported.

**Table S5.** GO Slim of differentially expressed genes between susceptible and DFB resistant *Culex pipiens*. P, biological process; F, molecular functions; C, cellular components.

**Table S6.** Gene Ontology enrichment analysis of up‐regulated genes between susceptible and DFB‐resistant *Culex pipiens* mosquitoes. BP, biological processes; CC, cellular components; MF, molecular functions.

**Table S7.** List of enrichment result GO annotation for up‐regulated DEGs of *Culex pipiens* after hypergeometric analysis.

## Data Availability

The data that support the findings of this study are available in the supplementary material of this article.
