# Supplementary material for: Comparative transcriptomics reveals different profiles between diflubenzuron‐resistant and ‐susceptible phenotypes of the mosquito Culex pipiens
Source: Pest Manag Sci. 2025 Feb 12;81(6):3370–7. doi: 10.1002/ps.8710 (PMC12074624; doi:10.1002/ps.8710)
Supplement: Supplementary file 1 — Figure S1. Bioinformatic workflow for transcriptomic analysis in Culex pipiens to identify differentially expressed genes (DEGs) associated with diflubenzuron (DFB) resistance. The process began with the analysis of both raw sequencing data (FASTQ) and a de novo transcriptome (unigenes) used as a reference (Mastrantonio et al., 2024). Transcripts were quantified using Salmon, both for transcriptome indexing and transcript quantification. Using the phenotype file (Phenodata.tsv) as input, the DEG list was generated by applying Iguaner (a tool based on DESeq2) and comparing DFB‐resistant and DFB‐susceptible samples. The results of DEGs were visualized as heatmaps and volcano plots. Open reading frames (ORFs) of DEGs were predicted using TransDecoder. Functional annotation was performed using EggNOG Mapper, and homology annotation was done with Diamond against databases (NR, Swiss‐Prot, and trEMBL). Outputs included functionally and homologously annotated DEGs, as well as functional enrichment results (e.g., gene set enrichment), also represented graphically. [file PS-81-3370-s007.docx]

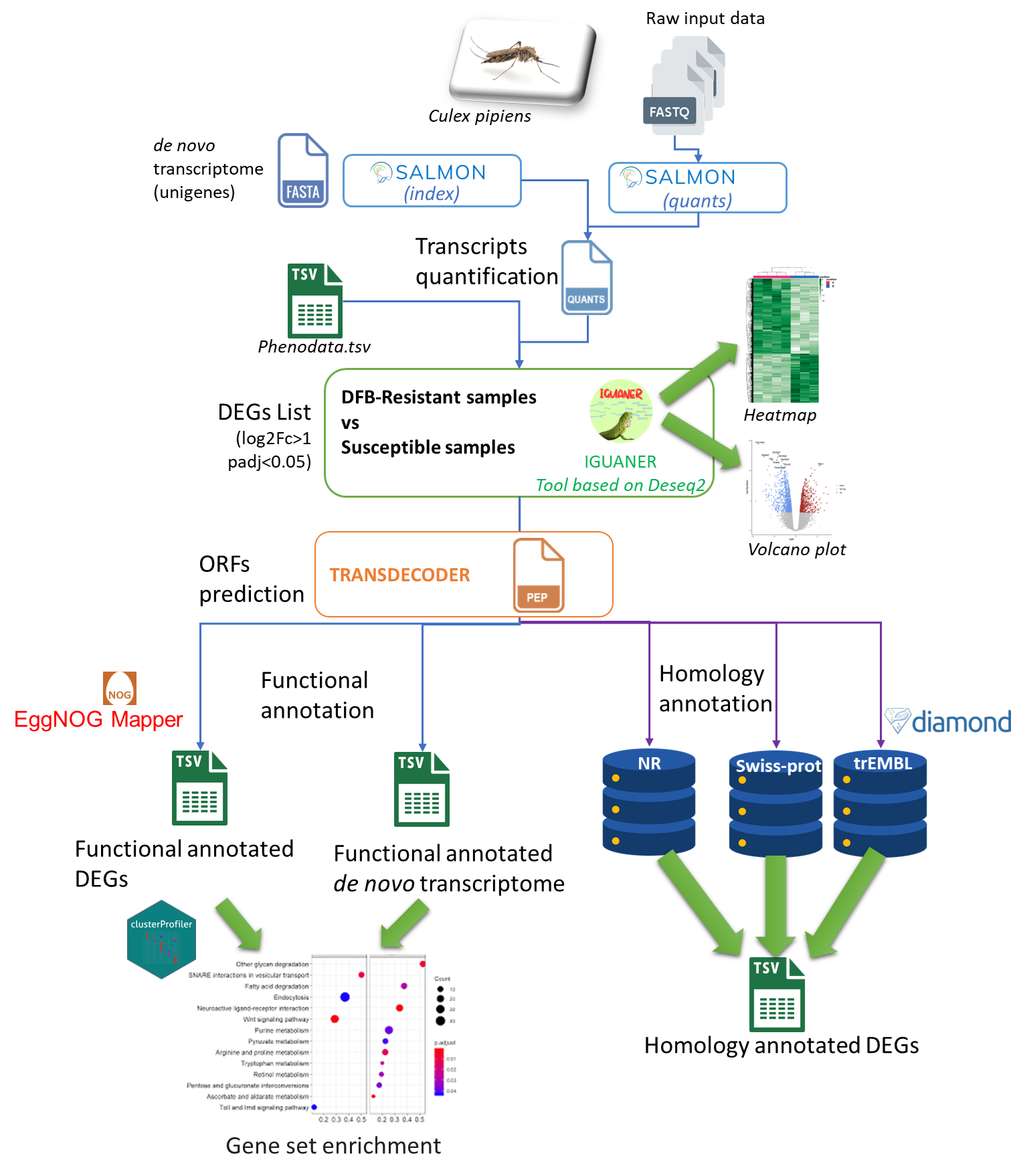


**Supplementary Figure 1.** Bioinformatic workflow for transcriptomic analysis in *Culex pipiens* to identify differentially expressed genes (DEGs) associated with Diflubenzuron (DFB) resistance. The process began with the analysis of both raw sequencing data (FASTQ) and a *de novo* transcriptome (unigenes) used as a reference (Mastrantonio et al. 2024). Transcripts were quantified using Salmon, both for transcriptome indexing and transcript quantification. Using the phenotype file (Phenodata.tsv) as input, the DEG list was generated by applying IGUANER (a tool based on DESeq2) and comparing DFB-resistant and DFB-susceptible samples. The results of DEGs were visualized as heatmaps and volcano plots. Open Reading Frames (ORFs) of DEGs were predicted using TransDecoder. Functional annotation was performed using EggNOG Mapper, and homology annotation was done with Diamond against databases (NR, Swiss-Prot, trEMBL). Outputs included functionally and homologously annotated DEGs, as well as functional enrichment results (e.g., gene set enrichment), also represented graphically.
