# Supplementary material for: Comparative transcriptomics reveals different profiles between diflubenzuron‐resistant and ‐susceptible phenotypes of the mosquito Culex pipiens
Source: Pest Manag Sci. 2025 Feb 12;81(6):3370–7. doi: 10.1002/ps.8710 (PMC12074624; doi:10.1002/ps.8710)
Supplement: Supplementary file 2 — Figure S2. Principal component analysis (PCA) shows the samples in the 2D plane spanned by their first two principal components. This type of plot is useful for visualizing the overall effect of experimental covariates and batch effects. [file PS-81-3370-s004.docx]

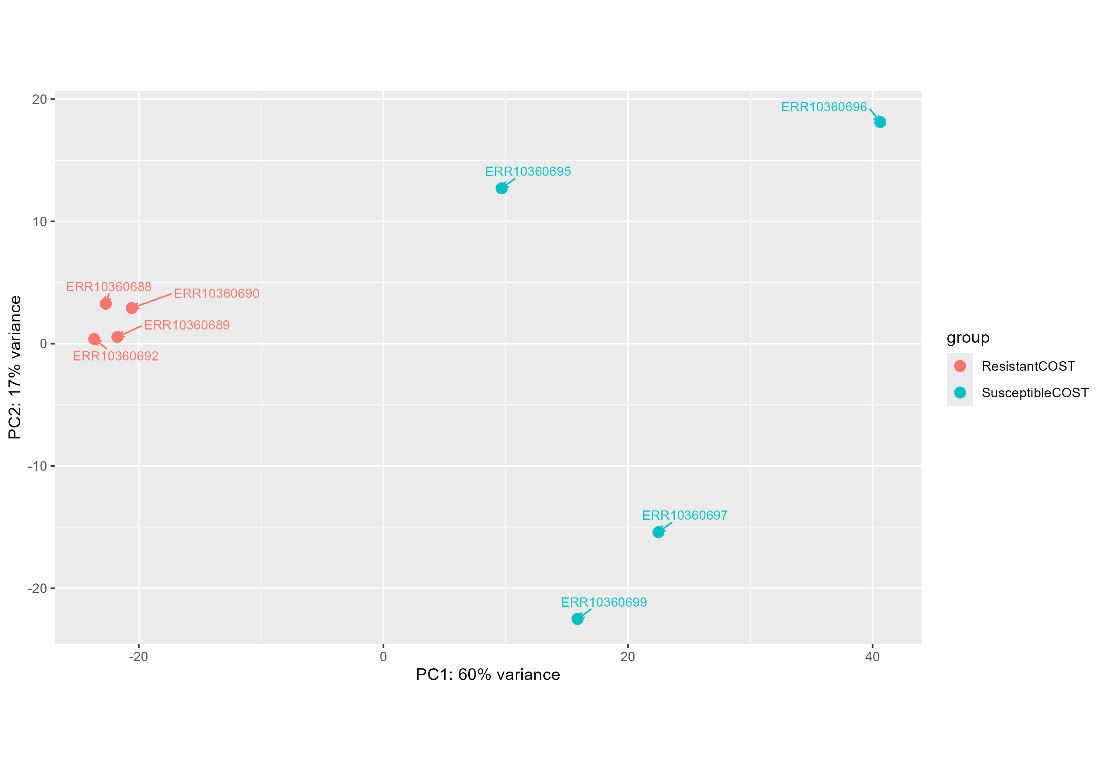


**Supplementary Figure 2.** Principal component analysis. PCA shows the samples in the 2D plane spanned by their first two principal components. This type of plot is useful for visualizing the overall effect of experimental covariates and batch effects.
