# Supplementary material for: Comparative transcriptomics reveals different profiles between diflubenzuron‐resistant and ‐susceptible phenotypes of the mosquito Culex pipiens
Source: Pest Manag Sci. 2025 Feb 12;81(6):3370–7. doi: 10.1002/ps.8710 (PMC12074624; doi:10.1002/ps.8710)
Supplement: Supplementary file 3 — Figure S3. Presentation of Gene Ontology (GO) classification. Histograms show the number of the differentially expressed transcripts assigned to GO terms – up‐regulated (A) and down‐regulated (B) – within three functional classes: biological processes, cellular components, and molecular functions. [file PS-81-3370-s001.docx]

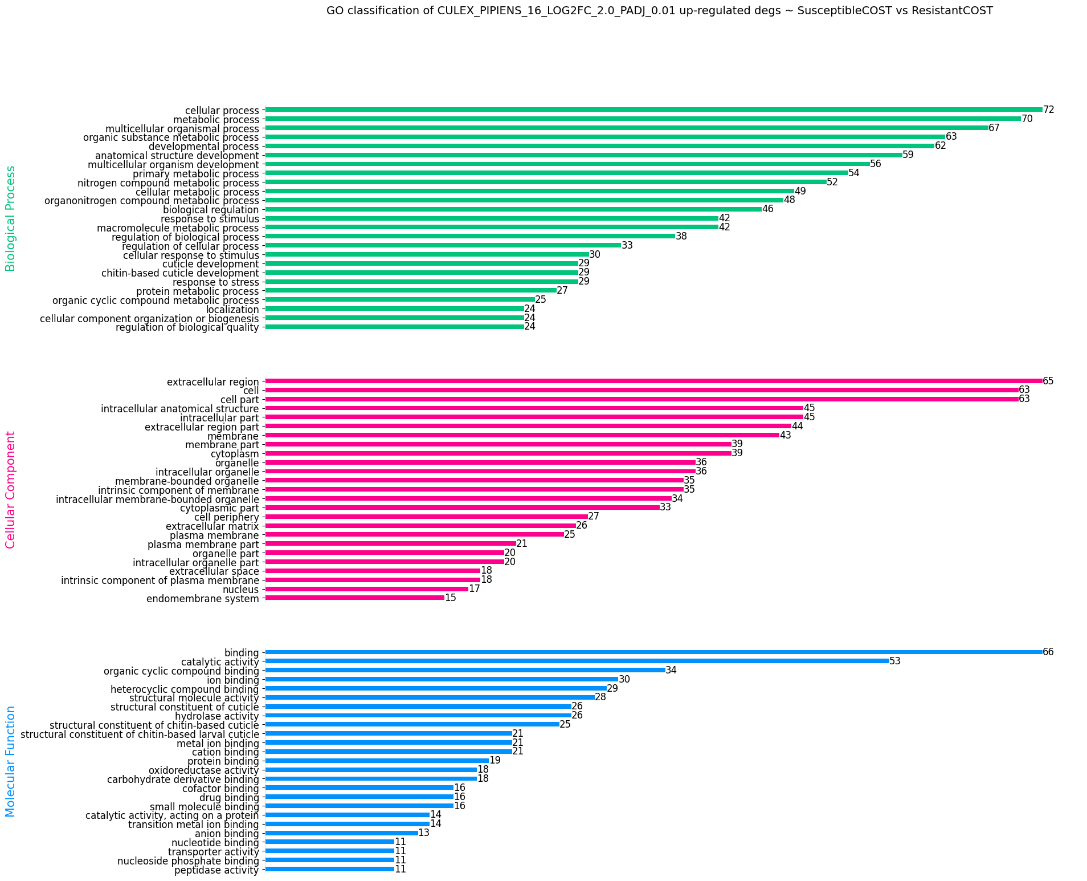

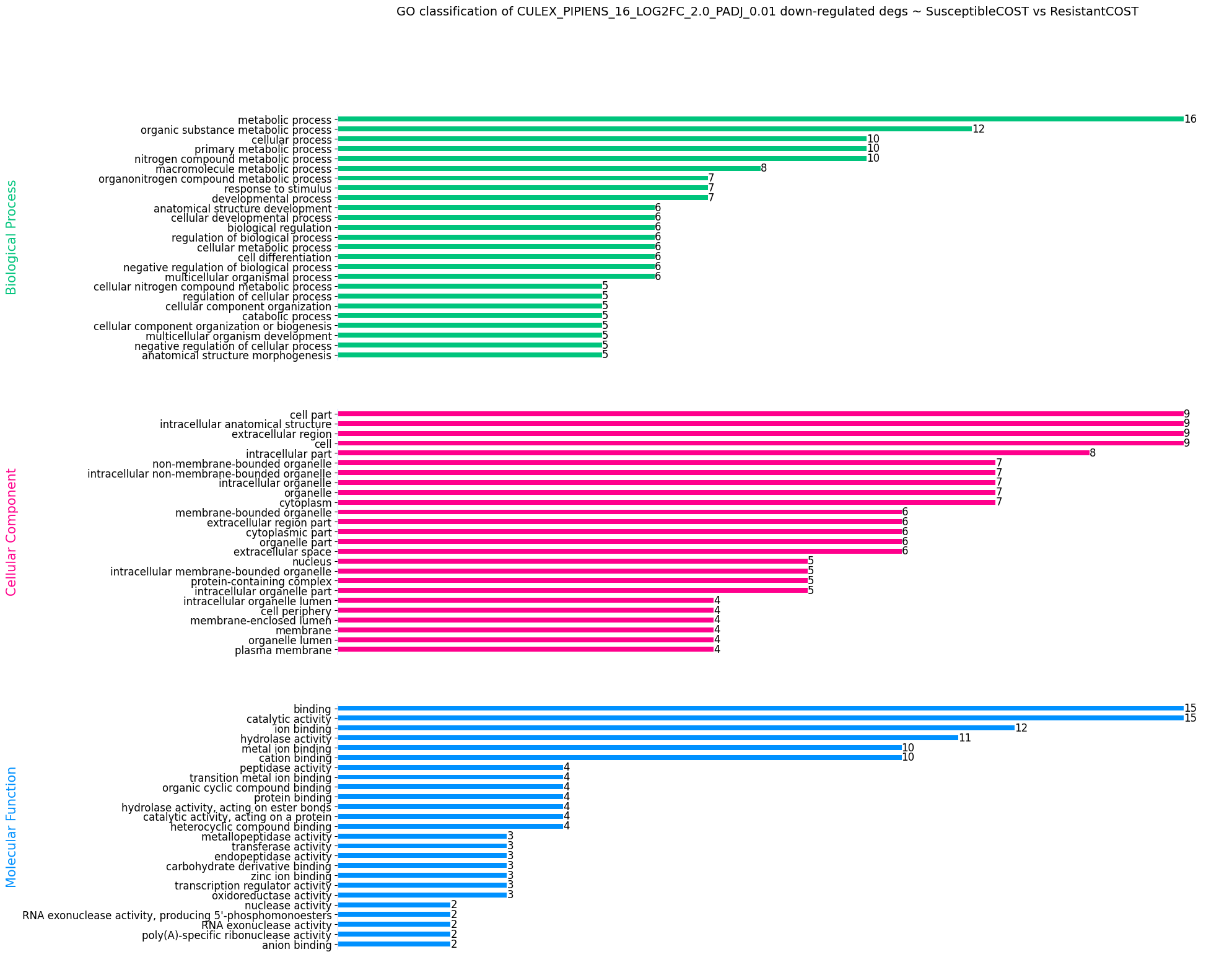


(A)

(B)

**Supplementary Figure 3**. Presentation of Gene ontology (GO) classification. Histograms show the number of the differentially expressed transcripts assigned to GO terms - UP-regulated (A) and DOWN-regulated (B) - within three functional classes: biological processes, cellular components and molecular functions.
