# Supplementary material for: Comparative transcriptomics reveals different profiles between diflubenzuron‐resistant and ‐susceptible phenotypes of the mosquito Culex pipiens
Source: Pest Manag Sci. 2025 Feb 12;81(6):3370–7. doi: 10.1002/ps.8710 (PMC12074624; doi:10.1002/ps.8710)
Supplement: Supplementary file 4 — Table S1. Summary data of the eight libraries deposited in the European Nucleotide Archive (ENA, BioProject: PRJEB47420). The sample code, strain condition, and the number of raw and trimmed reads per sample are shown. [file PS-81-3370-s008.docx]

**Supplementary Table 1.** Summary data of the eight libraries deposited in the European Nucleotide Archive (ENA, BioProject: PRJEB47420). The sample code, strain condition and the number of raw and trimmed reads per sample are shown.

| **Sample** | **Condition** | **Raw reads** | **Trimmed reads** |
| --- | --- | --- | --- |
| ERR10360695 | SusceptibleCOST | 68,850,032 | 62,820,900 (91%) |
| ERR10360696 | SusceptibleCOST | 76,598,300 | 68,146,132 (89%) |
| ERR10360697 | SusceptibleCOST | 72,653,248 | 62,903,628 (86%) |
| ERR10360699 | SusceptibleCOST | 49,575,476 | 38,883,708 (78%) |
| ERR10360688 | ResistantCOST | 75,657,372 | 65,583,016 (87%) |
| ERR10360689 | ResistantCOST | 83,210,660 | 76,111,468 (91%) |
| ERR10360690 | ResistantCOST | 83,669,916 | 69,666,436 (83%) |
| ERR10360692 | ResistantCOST | 76,949,748 | 65,731,840 (85%) |
