# Supplementary material for: Comparative transcriptomics reveals different profiles between diflubenzuron‐resistant and ‐susceptible phenotypes of the mosquito Culex pipiens
Source: Pest Manag Sci. 2025 Feb 12;81(6):3370–7. doi: 10.1002/ps.8710 (PMC12074624; doi:10.1002/ps.8710)
Supplement: Supplementary file 5 — Table S2. Overview of produced data files and their access on Figshare. [file PS-81-3370-s006.docx]

**Supplementary Table 2.** Overview of produced data files and their access on Figshare

| **Label** | **Name of data** | **File type** | **Data repository (URL)** |
| --- | --- | --- | --- |
| Data file 1 | *Culex pipiens* phenotypes | Text (*.txt) | [https://doi.org/](https://doi.org/10.6084/m9.figshare.27894762)10.6084/m9.figshare.28189013 |
| Data file 2 | *Culex pipiens* transcriptome | Fasta (*.fa) | [https://doi.org/](https://doi.org/10.6084/m9.figshare.27894762)10.6084/m9.figshare.22515256 |
| Data file 3 | *Culex pipiens* predicted ORFs of transcriptome | Fasta (*.pep) | [https://doi.org/](https://doi.org/10.6084/m9.figshare.27894789)10.6084/m9.figshare.28189256 |
| Data file 4 | *Culex pipiens* DEGs and ORFs | Fasta (*.fa) | [https://doi.org/](https://doi.org/10.6084/m9.figshare.27896766)10.6084/m9.figshare.28189265 |
| Data file 5 | *Culex pipiens* GO Functional Analysis | Folder | [https://doi.org/](https://doi.org/10.6084/m9.figshare.27894798) 10.6084/m9.figshare.28189268 |
| Image1 | Heatmap DEGs of defensome (with identifier) | Image *.png | [https://doi.org/](https://doi.org/10.6084/m9.figshare.27891237)10.6084/m9.figshare.28202669 |
